# DOCK2 deficiency due to a novel cryptic splice site mutation

**DOI:** 10.70962/jhi.20260146

**Published:** 2026-10-26

**Authors:** Manisha Ahuja, Ina C.D. Schim van der Loeff, Helen Griffin, Angela Grainger, Joseph D.P. Willet, Mary Slatter, Zohreh Nademi, Andrew R. Gennery, Andrew J. Cant, Su Han Lum, Sophie Hambleton, Karin R. Engelhardt

**Affiliations:** 1 Translational and Clinical Research Institute, Newcastle University, Newcastle upon Tyne, UK; 2 Clinical Immunology and Allergy, Newcastle upon Tyne Hospital NHS Foundation Trust, Newcastle upon Tyne, UK; 3 Children’s Haematopoietic Stem Cell Transplant Unit, Great North Children’s Hospital, Newcastle upon Tyne Hospital NHS Foundation Trust, Newcastle upon Tyne, UK

## Abstract

Autosomal recessive dedicator of cytokinesis 2 (DOCK2) deficiency causes severe, early-onset bacterial and viral infections. We report three siblings with DOCK2 deficiency due to a novel homozygous synonymous variant that results in cryptic mRNA splicing and absence of wild-type protein.

## Teaser

Three siblings with an immunodeficiency have a novel homozygous synonymous variant in *DOCK2*, which is predicted to not change the amino acid sequence. However, in silico analysis suggests a defect in mRNA splicing, which this study confirms. It results in a frameshift transcript and loss of DOCK2 protein expression, explaining the patients’ phenotype.

Patients with autosomal recessive dedicator of cytokinesis 2 (DOCK2) deficiency have been reported to present with early-onset severe viral and/or bacterial infections, including pneumonia, disseminated varicella, and mumps meningoencephalitis (1, 2). A prominent gastrointestinal phenotype is often observed, ranging from chronic diarrhea to severe colitis and in some cases complicated by fistula formation (1, 2). Immunologically, patients consistently demonstrate reduced T cell proliferation alongside CD4^+^, CD8^+^, and B cell lymphopenia and reduced natural killer (NK) cell degranulation (1). Humoral immunity is compromised, with impaired antibody responses, variably low IgG and sometimes elevated IgM and/or IgE levels (2). Thus far, autosomal recessive DOCK2 deficiency has been reported in 37 patients (2).

DOCK2 is a member of the Dock family of proteins and is primarily present in cells of hematopoietic origin, in particular lymphocytes (3). It functions as a guanine nucleotide exchange factor that activates Rac, a GTPase that is essential for actin polymerization and cytoskeletal rearrangements. This in turn is crucial for immune synapse formation and cellular adhesion and migration (3). DOCK2 is involved in T and B cell development, migration, and effector functions (1, 3). It also has a role in NK cell cytotoxicity (1) and regulates activation and migration in neutrophils and macrophages (3). DOCK2-deficient neutrophils exhibit impaired actin polymerization, reduced motility, and defective respiratory burst (3). These abnormalities likely contribute to ineffective microbial clearance and dysregulated inflammation at mucosal surfaces, thereby predisposing to intestinal pathology. Biopsies from two patients with colitis demonstrated focal neutrophil infiltrates (1). DOCK2 also regulates type 1 interferon production in plasmacytoid dendritic cells (1). *Dock2*^−/−^ mice recapitulate the human phenotype with T cell lymphopenia and loss of marginal zone B cells, near absence of Rac-mediated actin polymerization after chemokine stimulation, and defective lymphocyte migration (1, 3).

Recently, three distinct heterozygous missense variants in the *DOCK2* gene, resulting in reduced protein expression and impaired function, have been described in six patients (4). In contrast to patients with autosomal recessive complete loss of DOCK2, these patients with residual protein expression and a dominant-negative effect have a much milder phenotype of susceptibility to severe viral infections only and variable T cell lymphopenia and hypogammaglobulinemia.

**Figure 1.**
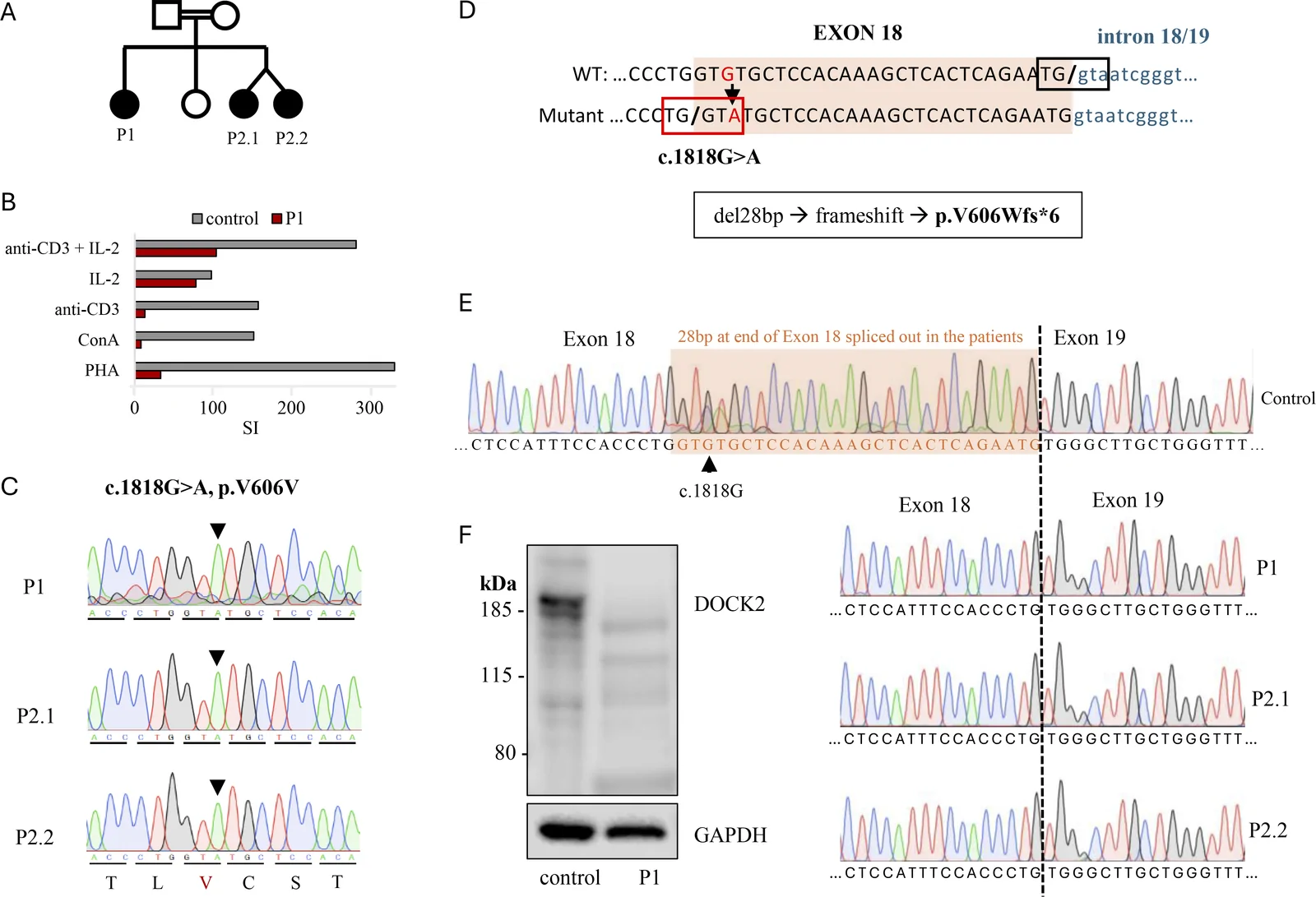
**Splicing defect in three siblings with S(CID) due to c.1818G>A variant in *DOCK2* creating a cryptic splice site. (A)** Pedigree. Square: male, circle: female. Double line indicates consanguinity. Filled symbol: affected. P2.1 and P2.2 are dizygotic twins. **(B)** Proliferation of T cells from control (grey bars) and P1 (red bars) against mitogens phytohemagglutinin (PHA), Concanavalin A (ConA), anti-CD3, and IL2. Proliferation is measured as cpm (counts per minute of tritiated thymidine). Shown is the stimulation index (SI), calculated as stimulated cpm/unstimulated cpm. **(C)** Capillary sequencing trace of genomic DNA confirming the homozygous c.1818G>A change in each patient. P1 underwent whole exome and whole genome sequencing. All three patients underwent capillary sequencing using both forward and reverse primers to confirm findings (*n* = 1). ClinVar accession number: SCV007666057. **(D)** Schematic of new cryptic splice donor site within exon 18. Nucleotides contributing to the splice donor site are boxed (black: wild-type splice site; red: cryptic splice site). The mutated base (c.1818G>A) is highlighted in red. The 28 bp that are spliced out in the patient are highlighted by orange shading. Aberrant splicing leads to a frameshift and premature stop codon: p.V606Wfs*6. **(E)** Sanger sequencing of cDNA obtained from patients reveals a splicing defect due to usage of a new cryptic splice donor site, removing the last 28 bp of exon 18, which contain the mutation site. **(F)** Immunoblotting of T lymphoblast lysates of control and P1, separated by SDS-PAGE and probed with an anti-DOCK2 antibody, showing lack of full-length DOCK2 protein expression in the index patient. Loading control: GAPDH. Due to limited patient material, one experiment was carried out using two P1 technical replicates. SCID, severe combined immunodeficiency; GAPDH, glyceraldehyde 3-phosphate dehydrogenase; SDS-PAGE, sodium dodecyl sulfate-polyacrylamide gel electrophoresis. Source data are available for this figure: SourceData F1.

In this report, we describe a novel *DOCK2* variant affecting three siblings who presented with a spectrum of disease ranging from combined immunodeficiency to severe combined immunodeficiency (Table 1). P1 was the first child born to consanguineous parents of Pakistani origin (Fig. 1 A) following a normal pregnancy and uncomplicated delivery (birth weight 2.85 kg). Other than maternal hypothyroidism and paternal alpha thalassemia carrier status there was no significant family history. P1 initially presented to pediatric services as a contact of a patient with pleural tuberculosis (TB), prompting anti-tuberculous treatment and Bacillus Calmette-Guérin vaccination. She subsequently had episodes of diarrhea which were initially treated as possible cow’s milk protein intolerance. A few months later, at 12 mo of age, she presented as an emergency with fever, weight loss, and ongoing diarrhea having spent 2½ mo in Pakistan. She was found to have CMV viremia and colitis and was treated with valganciclovir. Immunological workup (Table 1) revealed CD4^+^ T cell lymphopenia, absent naïve T cells and impaired T cell proliferation to PHA, ConA, and anti-CD3 (normal response to IL2) (Fig. 1 B). She had normal B and NK cell numbers, but B cells subsets showed <1% class switched, 7% memory, and 91% naïve cells. She had poor responses to vaccines against *Streptococcus pneumoniae* (4–9 mg/L; range 20–200 mg/L) and *Haemophilus influenzae* B (0.5–0.3 mg/L; range 1–20 mg/L), failing to boost after repeat vaccination. Her IgE was 270 IU/L (range 0–60 IU/L). A contemporary genetic workup was negative.

**Table 1.** Immunology investigations at presentation and details of transplantation

​	P1	P2.1	P2.2
Diagnosis	CID	Atypical SCID	Atypical SCID
Stem cell source	9/10 HLA-DQ mismatched unrelated PBSC	Mismatched unrelated TCRαβ/CD19-depleted PBSC Addback CD45RO^+^ memory T D+7	Mismatched unrelated TCRαβ/CD19-depleted PBSC Addback CD45RO^+^ memory T D+7
Conditioning	Campath 0.2 mg/kg Treosulfan 14 g/m^2^ Fludabarine 30 mg/m^2^	Treosulfan 30 g/m^2^ Fludabarine 160 mg/m^2^ Thiotepa 10 mg/kg ATG Grafalon 15 mg/kg Rituximab 200 mg/m^2^	Treosulfan 36 g/m^2^ Fludabarine 160 mg/m^2^ Thiotepa 10 mg/kg ATG Grafalon 15 mg/kg Rituximab 200 mg/m^2^
GVHD prophylaxis	CyA and MMF	CyA	CyA
Chimerism (most recent)	100%	100%	-
Age at transplant	15 mo	5 mo	14 mo

​	P1		P2.1		P2.2	
**Age at assessment**	**1 year**	**12 years**	**6 days**	**17 mo**	**6 days**	**17 mo**
Lymphocytes (cells/μl)	8,195	3,074	**1,612**	5,105	**736**	**1,007**
T cells CD3^+^ (cells/μl)	3,976	2,162	**604**	3,757	**361**	**682**
B cells (cells/μl)	2,244	608	385	1,100	119	**0**
NK cells (cells/μl)	1,566	281	551	208	223	307
CD4^+^ T cells (cells/μl)	**308**	1,042	**191**	2,060	**149**	**192**
CD8^+^ T cells (cells µl)	3,342	810	342	1,172	**128**	**74**
CD4:CD8	**0.09**	1.29	**0.56**	1.76	1.16	2.61
CD4^+^ naïve T cells (% of CD3^+^ cells)	**0**	**19% (411)**	**5% (30)**	**35% (1,315)**	**8% (29)**	**3% (20)**
CD8^+^ naïve T cells (% of CD3^+^ cells)	**<1%**	**15% (324)**	**5% (30)**	**10% (376)**	**9% (32)**	**3% (20)**
CD8^+^ effector T cells (% of CD3^+^ cells)	**4% (159)**	9% (195)	**0**	15% (564)	**0**	**3% (20)**
Naïve B cells (% of CD19^+^ cells)	**91%**	**74%**	​	89%	​	​
Memory B cells (% of CD19^+^ cells)	**7%**	**10%**	​	9%	​	​
Class-switched B cells (% of CD19^+^ cells)	**<1%**	13%	​	2%	​	​
IgG (g/L)	11.2	9.3	6.4 (1 mo)	6.7	6.2 (1 mo)	5.4
IgA (g/L)	0.06	1.82	0.09 (1 mo)	0.26	0.08 (1 mo)	<0.05
IgM (g/L)	0.26	0.92	0.99 (1 mo)	0.30	0.06 (1 mo)	0.09
Donor chimerism at last follow-up	100%		100%		100%	
Age at last follow-up	12 years		17 mo		17 mo	
Most recent clinical status	Alive and well		Alive and well		Alive and well	

Highlighted in bold are deviations from the normal reference ranges: CD3^+^ T cells (cells/μl)—neonatal: 600–5,000; 9–24 mo: 1,400–8,000; 10–16 years: 800–3,500. CD4^+^ T cells (cells/μl)—neonatal: 400–3,500; 9–24 mo: 900–5,500; 10–16 years: 400–2,100. CD8^+^ T cells (cells/μl)—neonatal: 200–1,900; 9–24 mo: 400–2,300; 10–16 years: 200–1,200. CD4/CD8 ratio—neonatal: 1.0–2.6; 9–24 mo: 0.9–3.9; 10–16 years: 0.9–3.4. CD19^+^ B cells—neonatal: 40–1,100; 9–24 mo: 600–3,100; 10–16 years: 200–600. CD15/CD56^+^ NK cells—neonatal: 100–1,900; 9–24 mo: 100–1,400; 10–16 years: 70–1,200. Example reference ranges for additional T and B cell subsets are available in literature (5). Gating strategy: CD4^+^CD45RA^+^CD27^+^ (CD4^+^ naïve T cells), CD4^−^CD45RA^+^CD27^+^ (CD8^+^ naïve T cells), CD4^−^CD45RA^+^CD27^−^ (CD8^+^ effector T cells), CD27^−^IgM^+^IgD^+^ (naïve B cells), CD27^+^IgM^+^IgD^+^ (memory B cells), CD27^+^IgM^−^IgD^−^ (class-switched B cells). (S)CID, (severe) combined immunodeficiency; GvHD, graft versus host disease; PBSC, peripheral blood stem cell; CyA, ciclosporin; MMF, mycophenolate mofetil; ATG, anti-thymocyte globulin.

P1 was started on antimicrobial prophylaxis and immunoglobulin replacement, and her nutrition was optimized before undergoing a (9/10) mismatched unrelated hematopoietic stem cell transplant (HSCT) at 15 mo of age (Table 1). Her recovery was complicated by mucositis, CMV reactivation (treated with foscarnet), and norovirus gastroenteritis, all of which resolved. Immunoglobulin replacement was stopped 1 year following HSCT. At latest clinical review, 10 years and 9 mo after HSCT, her donor chimerism was 100%. She was thriving with normal lymphocyte subsets (CD3: 2,162 cells/μl, CD4: 1,042 cells/μl, and CD8: 810 cells/μl) and protective vaccine responses (pneumococcal antibody 80 mg/L, *Haemophilus influenzae* B 1.92 mg/L, and tetanus toxoid antibody 0.74 IU/ml [range 0.1–10 IU/ml]).

P1 has one healthy sister and affected dizygotic twin sisters, P2.1 and P2.2 (Fig. 1 A), born at 37 wk of gestation by elective cesarean delivery due to maternal gestational diabetes. Each twin weighed 2.6 kg and both had significant T cell lymphopenia at birth. Furthermore, P2.1 had congenital CMV treated preemptively with valganciclovir (normal magnetic resonance imaging, normal hearing, and no evidence of eye disease). Both twins received haploidentical HSCTs (Table 1) with prior serotherapy to eliminate host immunity, prevent rejection, and reduce the risk of graft versus host disease in line with recently updated European Society for Blood and Marrow Transplantation/European Society for immunodeficiencies guidelines. P2.1 received a mismatched unrelated TCRαβ/CD19-depleted peripheral blood stem cell (PBSC) HSCT aged 5 mo with few complications. She had CD45RO+ memory T cell addback day (D) +7 after transplant. Her CMV reactivated D+15 but was successfully treated with ganciclovir. At last clinical review, 1 year after transplant, she is thriving. Her lymphocyte subsets are normal (CD3: 5,105 cells/μl, CD4: 2,060 cells/μl, and CD8: 1,172 cells/μl,) with naïve T cells present. She has stopped antiviral and immunoglobulin prophylaxis but remains on oral antibacterial prophylaxis. P2.2 similarly underwent mismatched unrelated TCRαβ/CD19-depleted PBSC HSCT with CD45RO^+^ memory T cell addback. Her transplant was delayed due to donor availability and respiratory isolates of influenza and respiratory syncytial virus that were treated with oseltamivir and palivizumab. At last clinical review, D+98 after HSCT, she was clinically well, has neutrophil and NK cell engrafted but remains on antimicrobial and immunoglobulin prophylaxis. Given the relatively short duration of follow-up, longer term immune reconstitution and transplant outcome remain to be established.

Illumina short-read exome and genome sequencing in P1 identified a homozygous, synonymous single-nucleotide variant in *DOCK2* at NC_000005.10: g.169714186G>A; NM_004946.3: c.1818G>A (Fig. 1 C). This variant is absent from the gnomAD v4 population database, and parents were both heterozygous carriers (data not shown). Although simple in silico translation predicted no change in the amino acid sequence, the spliceAI predicted score for this variant was donor gain: delta score = 0.86 and delta position = −3 bp. This predicts with high likelihood (∼86% chance of experimental validation) that a new damaging, cryptic, and exonic splice donor site will be created 3 bp upstream from the c.1818G>A variant (Fig. 1 D). In turn, this could be expected to result in substantial truncation of any stable DOCK2 protein product due to homozygous frameshift (p.V606Wfs*6) and/or reduced protein synthesis due to nonsense-mediated decay of the variant mRNA.

To test this prediction of altered splicing, we isolated RNA from dermal fibroblast cultures established from P1, generated cDNA, and amplified exons 17–19 by PCR. Sanger sequencing of targeted amplicons indeed revealed a 28-bp deletion at the end of exon 18 (c.1816_1843del) due to cryptic splicing with no residual normal splicing evident in any of the three patients (Fig. 1 E). To confirm this, T lymphoblast lysates from P1 were separated by sodium dodecyl sulfate–polyacrylamide gel electrophoresis and probed with an anti-DOCK2 antibody. Glyceraldehyde 3-phosphate dehydrogenase was used as a loading control. This revealed an absence of full-length DOCK2 protein in the patient samples, which was present in a control at the expected molecular weight (Fig. 1 F). These findings align with previously reported DOCK2-deficient patients, in whom absent or markedly reduced protein expression underpins disease pathogenesis. P2.1 and P2.2 were subsequently screened at birth for the same defects as P1. Capillary sequencing of gDNA and cDNA confirmed presence of the synonymous variant (Fig. 1 C) and aberrant splicing (Fig. 1 E).

The clinical and immunological phenotype observed in our patients, including early-onset viral infection, gastrointestinal involvement, humoral immunodeficiency, T cell lymphopenia, and impaired T cell proliferation, is consistent with the established spectrum of DOCK2 deficiency. This concordance further supports the pathogenicity of this variant. Our findings emphasize the need for careful evaluation of rare synonymous variants of relevant genes in patients with strong phenotypic suspicion of monogenic disease. Integration of in silico splice prediction tools, segregation analysis, and functional validation are critical in establishing pathogenicity. As access to genomic sequencing continues to expand, improved recognition and interpretation of these “non-silent” synonymous variants will be essential to maximize diagnostic yield and guide appropriate clinical management, including timely curative interventions such as hematopoietic stem cell transplantation.

## Ethics statement

The patients’ family gave written consent for approved research including data collection, analysis, and publication (Newcastle and North Tyneside 1 Research Ethics Committee, REC reference 20/NE/0044).

## Supplementary Material

SourceData F1 is the source file for Fig. 1.

## Data Availability

Data are available in the article itself. Expanded immunoblots from Fig. 1 G have been submitted as source data. The identified variant NC_000005.10: g.169714186G>A; NM_004946.3: c.1818G>A (Fig. 1 D) has been submitted to ClinVar (accession number: SCV007666057).
