# Supplementary material for: DOCK2 deficiency due to a novel cryptic splice site mutation
Source: J Hum Immun. 2026 Oct 26;3(1):e20260146. doi: 10.70962/jhi.20260146 (PMC13701129; doi:10.70962/jhi.20260146)

1) Figure 1G

Nitrocellulose membrane split for staining with Anti-DOCK2 antibody (A) and Anti-GAPDH antibody (B).

- P1 denotes patient sample – two technical replicates
- C1 is used in the manuscript figure
- L1 and L2 represent two different molecular weight ladders
- C2 – C4 represent control T lymphoblasts that were not incorporated into manuscript figure as these lysates had degraded following prolonged cryopreservation (no gapdh or dock2 staining seen) and were therefore discarded.
- Red outline demonstrates regions of blot cropped for manuscript figure.

A

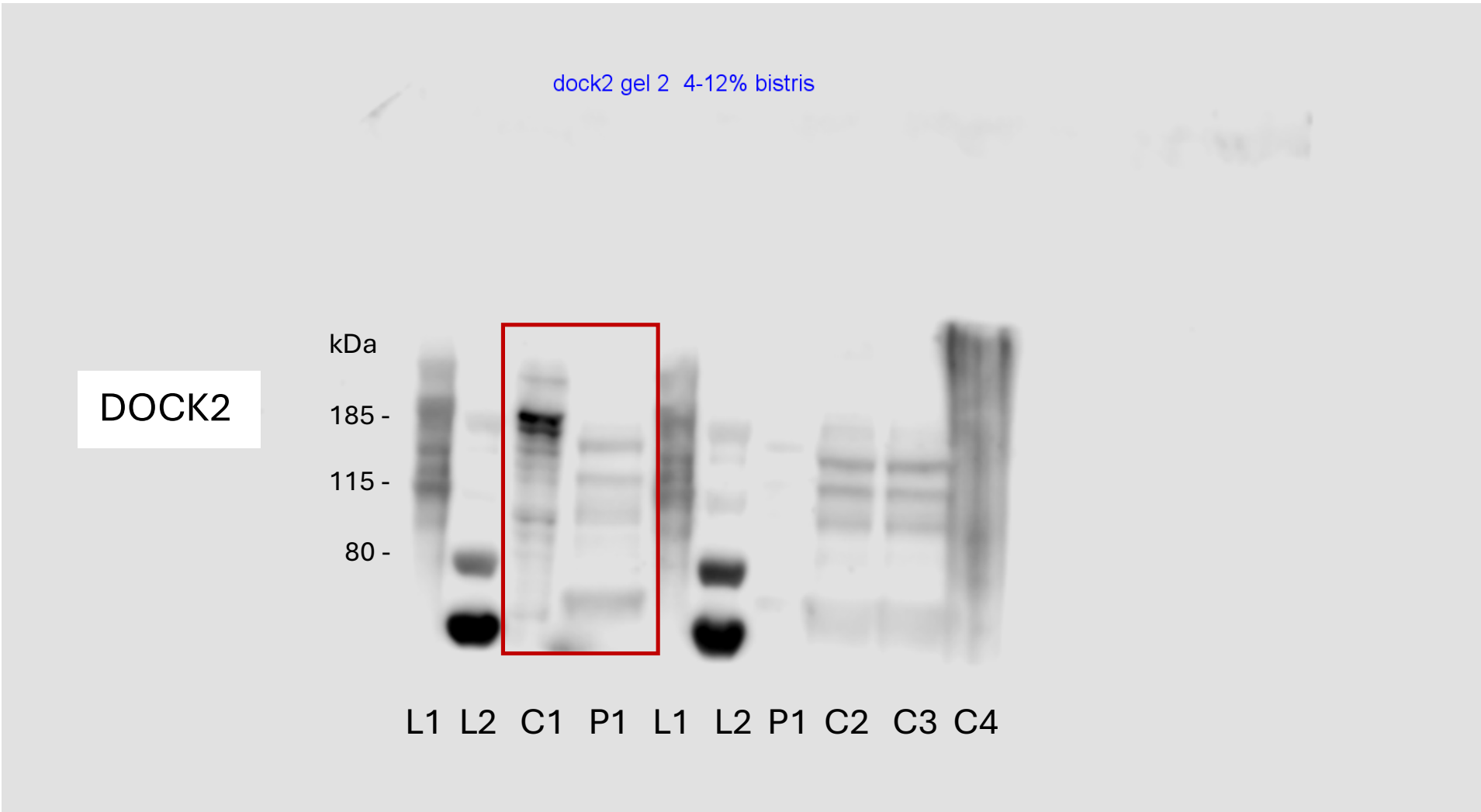

B

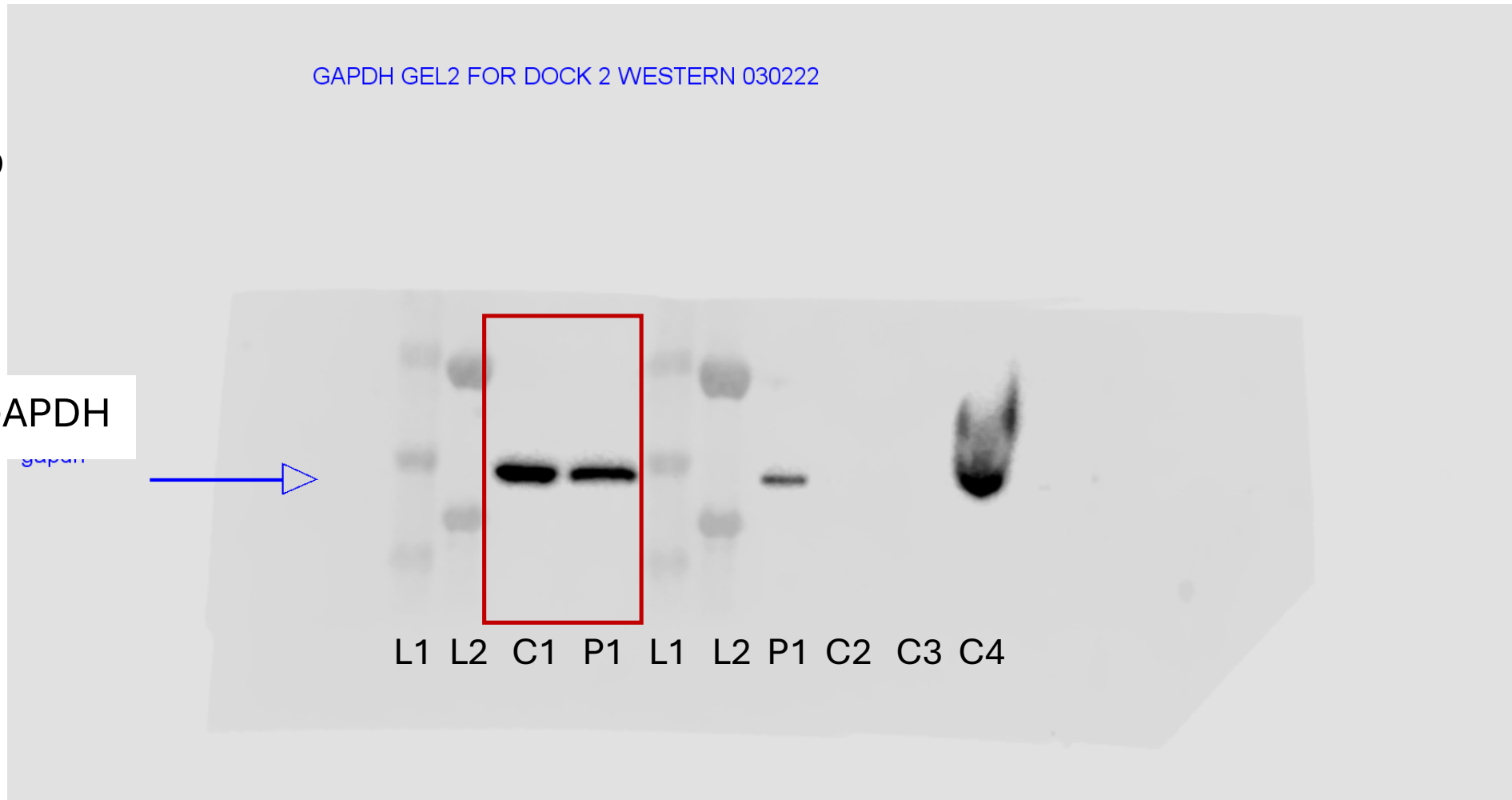

Supplement: Supplementary file 1 [file jhi_20260146_sourcedataf1.pdf]
